# TNF-α induces AQP4 overexpression in astrocytes through the NF-κB pathway causing cellular edema and apoptosis

**DOI:** 10.1042/BSR20212224

**Published:** 2022-03-17

**Authors:** Hong Lu, Li Ai, Baoyue Zhang

**Affiliations:** Department of Medical Imaging, The Seventh People’s Hospital of Chongqing, Chongqing 400054, P.R. China

**Keywords:** AQP4, astrocytes, NF-κB, TNF-α

## Abstract

Aquaporin 4 (AQP4) is highly expressed on astrocytes and is critical for controlling brain water transport in neurological diseases. Tumor necrosis factor (TNF)-α is a common cytokine found in disease microenvironment. The aim of the present study was to determine whether TNF-α can regulate the expression of AQP4 in astrocytes. Primary astrocyte cultures were treated with different concentrations of TNF-α and the cell viability was assessed through cell counting kit-8 (CCK-8) assay and AQP4 expression was detected by qPCR, Western blots, and immunofluorescence assays. The activation of nuclear factor κ-light-chain-enhancer of activated B cells (NF-κB) pathway was detected by Western blot. Further, dual-luciferase reporting system and chromatin immunoprecipitation (ChIP) were used to detect the transcriptional regulation of AQP4 by p65. These experiments demonstrated that treatment with TNF-α can lead to astrocyte edema and an increase in AQP4 expression. Following TNF-α treatment, the expression levels of P-IKKα/β-IκBα and P-p65 increased significantly over time. The results of the dual-luciferase reporter system and ChIP assays revealed that p65 protein and AQP4 promoter had a robust binding effect after TNF-α treatment, and the NF-κB pathway inhibitor, BAY 11-7082 could inhibit these effects of TNF-α. The expression level of AQP4 was significantly decreased upon p65 interference, while the astrocyte viability was significantly increased compared with that in the TNF-α only group. In conclusion, TNF-α activated NF-κB pathway, which promoted the binding of p65 to the AQP4 gene promoter region, and enhanced AQP4 expression, ultimately reducing astrocyte viability and causing cell edema.

## Introduction

Cerebral hemorrhage refers to non-traumatic intraparenchymal bleeding. Brain damage caused by cerebral hemorrhage comprises direct compression of hematoma and secondary brain injury. Secondary cerebral edema is an important cause of neurological dysfunction and death, and an essential factor affecting the prognosis of patients. The current clinical treatment of cerebral edema includes mainly surgical removal of hematoma, dehydration, and glucocorticoid therapy. However, the therapeutic effect can barely meet the satisfaction of patients. Therefore, finding a quick and effective treatment method for cerebral edema is one of the current research hotspots [1].

Aquaporin 4 (AQP4) is found on cell membranes and is involved in regulating the water content in cells. The astrocyte membrane contains various ion channels, AQP4, and amino acid transporters. When a number of ions and neurotransmitters are ingested, the intracellular osmotic pressure increases, due to which water enters the cell, triggering a transient increase in cell volume, thereby activating the astrocyte volume regulation mechanism [2,3]. The enlarged cell volume activates a series of physiological reactions, triggering cells to discharge various substances and water to restore normal cell volume. Under normal physiological conditions, with neural activity, the astrocyte podocytic process presents a transient increase in volume, however, when the cell volume regulation mechanism is impaired, cell edema may develop.

Several studies have demonstrated that the level of AQP4 expression is up-regulated in cytotoxic cerebral edema, and when AQP4 expression is down-regulated, the brain edema is reduced [4,5]. The phenotypes of transgenic mice lacking AQP4 function in cerebral water homeostasis, astrocyte migration, and neuronal signal transduction have been examined. In cytotoxic cerebral edema models such as water intoxication, localized cerebral ischemia, and bacterial meningitis, AQP4-null animals showed lower brain swelling and improved neurological outcomes. AQP4-null animals in models of vasogenic edema, including cortical freeze injury, brain tumor, brain abscess, and hydrocephalus, show brain swelling and the clinical prognosis is poor. This is likely due to reduced AQP4-dependent brain water clearance [6]. An increased brain water accumulation and intracranial pressure were observed in AQP4-null mice compared with wildtype mice with brain tumors, brain abscesses, focal cortical freeze injury, and after direct infusion of normal saline into brain extracellular space. This indicates that vasogenic edema fluid is eliminated via an AQP4-dependent pathway. Moreover, AQP4-null animals develop more severe hydrocephalus than wildtype mice in a kaolin injection model of obstructive hydrocephalus; it is most likely due to decreased water clearance through the ependymal blood–brain barriers in AQP4-null mice [7].

Nuclear factor κ-light-chain-enhancer of activated B cells (NF-κB) is a crucial transcription factor that regulates immune and inflammatory responses, and overactivated NF-κB is at the center of cerebral ischemia and edema reaction. NF-κB belongs to the Rel protein family, and its members mainly include NF-κB1 (p50/p105), NF-κB2 (p52/p100), RelA (p65), RelB, and c-Rel. Different members of this family can together form homo- or heterodimers. The classical dimer consists of p65 and p50, which are widely found in eukaryotes. In the resting state, NF-κB binds to its inhibitory protein, inhibitor of κB (IκB) and remains in the cytoplasm. After cerebral ischemic injury, it activates the IκB kinase (IKK) complex. After IKKs are activated, they induce IκBα phosphorylation and continue to degrade and dissociate from NF-κB, and then rapidly translocateNF-κB to the nucleus to specifically bind to the promoters of its target genes, and activate their transcription. The target genes include cytokines, namely tumor necrosis factor-α (TNF-α), adhesion molecules, and chemokines related to inflammation. On the other hand, the products regulated by NF-κB, including TNF-α, can further activate NF-κB, forming a complex positive feedback regulation loop that can amplify and continue the inflammatory response [8,9].

NF-κB can be activated in response to a variety of stimuli, including cytokines (TNF-α, IL-1β), growth factors (epidermal growth factor), bacterial and viral products (lipopolysaccharide, dsRNA), ultraviolet and ionizing radiations, reactive oxygen species (ROS), and DNA damage and oncogenic stress originating from within the cells. Almost all stimuli ultimately activate a large cytoplasmic protein complexes, the IκB and IKK via a so-called ‘canonical pathway’ [10]. TNF-α presents many inflammatory factors around the hemorrhage and in blood after cerebral hemorrhage. Multiple studies have shown that TNF-α participates in the formation and development of cytotoxic brain edema and can up-regulate the expression of AQP4 [11,12]. However, the regulatory effect of TNF-α on astrocytes and the specific molecular mechanism are still unclear.

Therefore, the present study aimed to explore mechanism by which TNF-α regulates astrocyte viability and edema, specifically the involvement of the NF-κB signaling pathway and AQP4 protein levels in astrocytes, since elucidating this mechanism might identify novel targets for the treatment of cerebral edema after cerebral hemorrhage.

## Materials and methods

### Primary culture of astrocytes

The animals used in the present study were housed in the animal facility located in the laboratory of Department of Medical Imaging of The Seventh People’s Hospital of Chongqing. None of the animal experiments involved the use of anesthesia. Suckling Sprague–Dawley rats within 48 h of birth (Experimental Animal Center of Army Medical University) were decapitated and their brains were removed. Meninges and hippocampus were stripped, washed with phosphate-buffered saline (PBS, C0221A, Beyotime, China) three-times, and then transferred to a disposable 60-mm² cell culture dish and cut into pieces. A 0.25% pancreatin (A600682, Sangon, China) solution was added to the samples to form a suspension and incubated for 8 min at 37°C with 5% CO_2_. The digestion was terminated using a complete medium containing 15% fetal bovine serum (SH30088.03, HyClone, U.S.A.), 2 mM l-glutamine (A600224, Sangon, China) and penicillin/streptomycin (B540732, Sangon, China), and filtered through a 200-cell sieve. The cell suspension was centrifuged, resuspended, inoculated into a cell culture flask, and cultured in an incubator for 7 days. When the cells were confluent, the flasks were placed on a constant temperature shaker (200 rpm) overnight. The cells at the bottom of the flask were harvested by digestion with Trypsin (A003702, Sangon, China), the cells were then centrifuged, resuspended, and seeded in a cell culture flask to obtain purified first-generation astrocytes [13,14]. The isolated cells were stained with glial fibrillary acidic protein (GFAP) (Supplementary Figure S1).

### TNF-α induces the expression of AQP4 gene in astrocytes

The second-passage astrocytes on day 1 were seeded into six-well plates at a concentration of 200000 cells in 2 ml per well. After the cells were confluent, the complete medium was replaced with a basic medium and the cells were starved for 24 h to achieve synchronization. The cells were treated with different concentrations of TNF-α (C600158, Sangon, China) [15,16]. After 6 h, RNA or protein was extracted for subsequent analyses. BAY 11-7082 (5 μM, S2913, Selleck, China) was used to inhibit the NF-κB signaling pathway, and 1% dimethylsulfoxide (DMSO) was used as control [17].

### Detection of cell viability by cell counting kit-8 kit

The second-passage astrocytes on day 1 were inoculated in a 96-well plate at a concentration of 20000 cells in 100 μl per well. After 48 h of culture, when the cells reached 80% confluence, they were starved for 24 h to achieve synchronization. The cells were treated with TNF-α or one of the controls. Next, by following the manufacturer’s instructions, cell counting kit-8 (CCK-8) reagent (C0037, Beyotime, China) was used to determine cell viability by measuring the optical density value of each well at 490 nm.

### Lentivirus packaging and cell infection

pLVX-IRES-mCherry was used to construct the lentiviral overexpression vector. The coding sequence (CDS) regions of AQP4 or p65 were cloned at the multiple cloning sites of the vector. pLVX-shRNA1-puro was used to construct the lentiviral interference vector, and the AQP4 interference sequence was GCATTCAATAAGTTACGGTTA, the p65 interference sequence was GCTGCGTATTGAAGATATTAA. Both vectors were synthesized by Beijing General Biotechnology Co., Ltd. AQP4 overexpression lentivirus, AQP4 interference lentivirus, p65 interference lentivirus, and control lentivirus were packaged by Chongqing Biomedicine Biotechnology Co., Ltd. The cell infection procedures were modified from a previously published protocol [18].

### AQP4 gene expression level

The TRIzol method (R0016, Beyotime, China) was applied to extract RNA. For reverse transcription of RNA into cDNA (RR037Q, TAKARA, Japan), the reaction system was as follows: a final volume (10 μl) reaction system containing, 5× PrimeScript RT Master Mix (2 μl), total RNA (2 μl), and RNase-free ddH_2_O (6 μl). The reaction conditions were set as follows: 37°C for 15 min, 85°C for 5 s, and 4°C for 5 min. The reaction volume for real-time fluorescent quantitative PCR (RR086A, TAKARA, Japan) was 25 μl comprising 2× SYBR Premix (12.5 μl), forward and reverse primers (1 μl each), cDNA (2 μl), and ddH_2_O (8.5 μl). The reaction conditions were as follows: 95°C for 30 s, 95°C for 5 s, 60°C for 30 s (40 cycles). Each sample had four replicate wells, with tubulin as the internal control. The 2^−ΔΔ_t_^ method was used to calculate the relative expression levels. The reference primer sequences were, forward primer: CGG TTC ATG GAA ACC TCA CT, and reverse primer CAT GCT GGC TCC GGT ATA AT.

### Western blot assay

After lysing astrocytes in radioimmunoprecipitation assay lysis buffer (P0013C, Beyotime, China), the lysate was centrifuged at 12000×***g*** for 30 min at 4°C and the supernatant was collected. After quantitatively balancing the histone concentrations, the sodium dodecyl sulfate (SDS) loading buffer was added, proteins were denatured at 95°C for 5 min, followed by electrophoresis on 10% SDS/PAGE gels (P0670, Beyotime, China), and then transferred to a nitrocellulose membrane. After blocking the nitrocellulose membrane (FFN08, Beyotime, China) with 5% bovine serum albumin (BSA, ST023, Beyotime, China) for 2 h, antibodies against P-IKKα/β (1:100, ab194528, Abcam, U.S.A.), IKKα (1:500, ab38515, Abcam, U.S.A.), IKKβ (1:500, ab124957, Abcam, U.S.A.), P-IκBα (1:100, ab133462, Abcam, U.S.A.), IκBα (1:500, ab32518, Abcam, U.S.A.), P-p65 (1:100, ab31624, Abcam, U.S.A.), p65 (1:500, ab32536, Abcam, U.S.A.), AQP4 (1:1000, ab259318, Abcam, U.S.A.), and tubulin (1:2000, ab7291, Abcam, U.S.A.) were added and incubated overnight at 4°C. The membrane was washed the next day, and horseradish peroxidase-labeled secondary antibodies (1:2000, ab288151, Abcam, U.S.A.) were added and incubated at room temperature for 2 h. After washing the membrane, the ECL developer was added, and the Gel Doc™ XR with an imaging system (Bio-Rad, U.S.A.) was used to record images.

### Immunofluorescence assay

The cells were plated on glass slides in the wells of a 24-well plate, and ∼90% of the cells covered the glass slide. The medium was removed and the slides were washed twice with 0.01 M PBS solution. The slides were transferred to another 24-well plate, and the cells were fixed in 4% paraformaldehyde (A500684, Sangon, China) for 30 min. After that, the paraformaldehyde was discarded, washed three-times with 0.01 M PBS, and 5% BSA blocking solution was added and blocked for 2 h. A 1:200 diluted primary antibody GFAP (1:50, ab7260, Abcam, U.S.A.) and AQP4 (1:50, ab259318, Abcam, U.S.A.) were added and incubated overnight at 4°C, then washed three-times with 0.01 M PBS on the following day, and a secondary antibody (1:500, ab150080, Abcam, U.S.A.) was added and placed on a shaker for 2 h at room temperature in the dark. Again, the slide was washed three-times with 0.01 M PBS, the slide was clamped out and mounted with mounting medium for microscopy.

### Detection of promoter activity by dual luciferase assay

A dual luciferase reporter vector with the AQP4 promoter sequence was constructed by Chongqing Biomedicine Biotechnology Co., Ltd. The reporter gene plasmid and pRL-TK (internal control plasmid) were co-transfected into the cells. During co-transfection, the transfection amount of reporter gene plasmid and the internal control plasmid was 10:1. Luciferase Assay Reagent II (LARII), the substrate of firefly luciferase was prepared. LARI was dissolved in LARII buffer. Following addition of 1× PLB, the cells were lysed at room temperature for 15 min, and the fluorescence values were measured. Utilizing the Stop&Glo, the substrate of *Renilla* luciferase was prepared. Ten microliters of cell lysate was added to 40 μl of LARII, pipetted, and mixed well, and the readings were checked, which was the firefly luciferase value. Then 40 μl Stop&Glo was added, and the readings were obtained again, which was the value of *Renilla* luciferase. First, each tube’s firefly luciferase/*Renilla* luciferase ratio was calculated. Then, the ratio of the control group was set as unit 1 to get the relative luciferase activity of different treatment groups and the regulation activity of gene transcription of the treatment group [19].

### Chromatin immunoprecipitation assay

Chromatin immunoprecipitation (ChIP) assay was carried out according to the instructions in the Promega kit (G9410, Promega, U.S.A.). First, cells grown on a 10-cm dish were fixed with 1% formaldehyde for 10 min at 37°C. Then 450 μl (2.5 M) of glycine was added to a Petri dish, mixed well, and placed at room temperature for 5 min. The medium was then removed, the cells were rinsed with ice-cold PBS twice, and were collected in a 15-ml centrifuge tube using a cell scraper. The cells were centrifuged at 2000 rpm for 5 min after precooling. The SDS lysis buffer was added to achieve a terminal concentration of 2 × 10^6^ cells per 200 μl along with 40 μl of protease inhibitor complex. The cells were lysed by ultrasonication using a sonicator (VCX750, SONICS, U.S.A.) at 25% power, 4.5 s shock, 9 s gap, for a total of 14-times, followed by centrifugation at 10000×***g*** at 4°C for 10 min. To 100 μl of the supernatant, 900 μl ChIP Dilution Buffer and 20 μl of 50× PIC were added. Then 60 μl of Protein A agarose and salmon sperm DNA mix was added to each tube and mixed at 4°C for 1 h. After 1 h, the tubes were allowed to stand for 10 min at 4°C and centrifuged at 700 rpm for 1 min. Twenty microliters of each supernatant was taken as input. One microliter of antibody was added to only one of the tubes and flipped at 4°C overnight. To 100 μl of supernatant post-sonication, 4 μl of 5 M NaCl was added and reverse cross-linking was performed at 65°C for 2 h. After that, 60 μl Protein A agarose/salmon sperm DNA mix was added to each tube and flipped at 4°C for 2 h. Tubes were allowed to stand at 4°C for 10 min, and then centrifugation was performed at 700 rpm for 1 min, and the supernatant was discarded. A 250-μl elution buffer was added into each tube, and the tubes were rotated at room temperature for 15 min. After standing and centrifuging, the supernatant was collected. To each tube, 20 μl of 5 M NaCl was added for a final NaCl concentration of 0.2 M and mixed well for cross-linking reversal at 65°C overnight. After unlinking, 1 μl RNaseA (MBI, Lithuania) was added to each tube and incubated at 37 °C for 1 h. Finally, a 10 μl of 0.5 M EDTA, 20 μl of 1 M Tris/HCl (pH 6.5), 2 μl of 10 mg/ml protease K were added to each tube and incubated at 45°C for 2 h. Omega glue was used to recycle the kit. The final sample was soluble in 100 μl ddH_2_O [20].

### Statistical methods

GraphPad Prism 8.0 (GraphPad Software, U.S.A.) statistical software was used for data analysis. Experimental data were expressed as mean ± standard deviation (x ± s) and one-way ANOVA was used to compare between groups. *P*<0.05 was considered statistically significant.

## Results

### Detection of cell viability and cell volume of astrocytes treated with different concentrations of TNF-α

**Figure 1:**
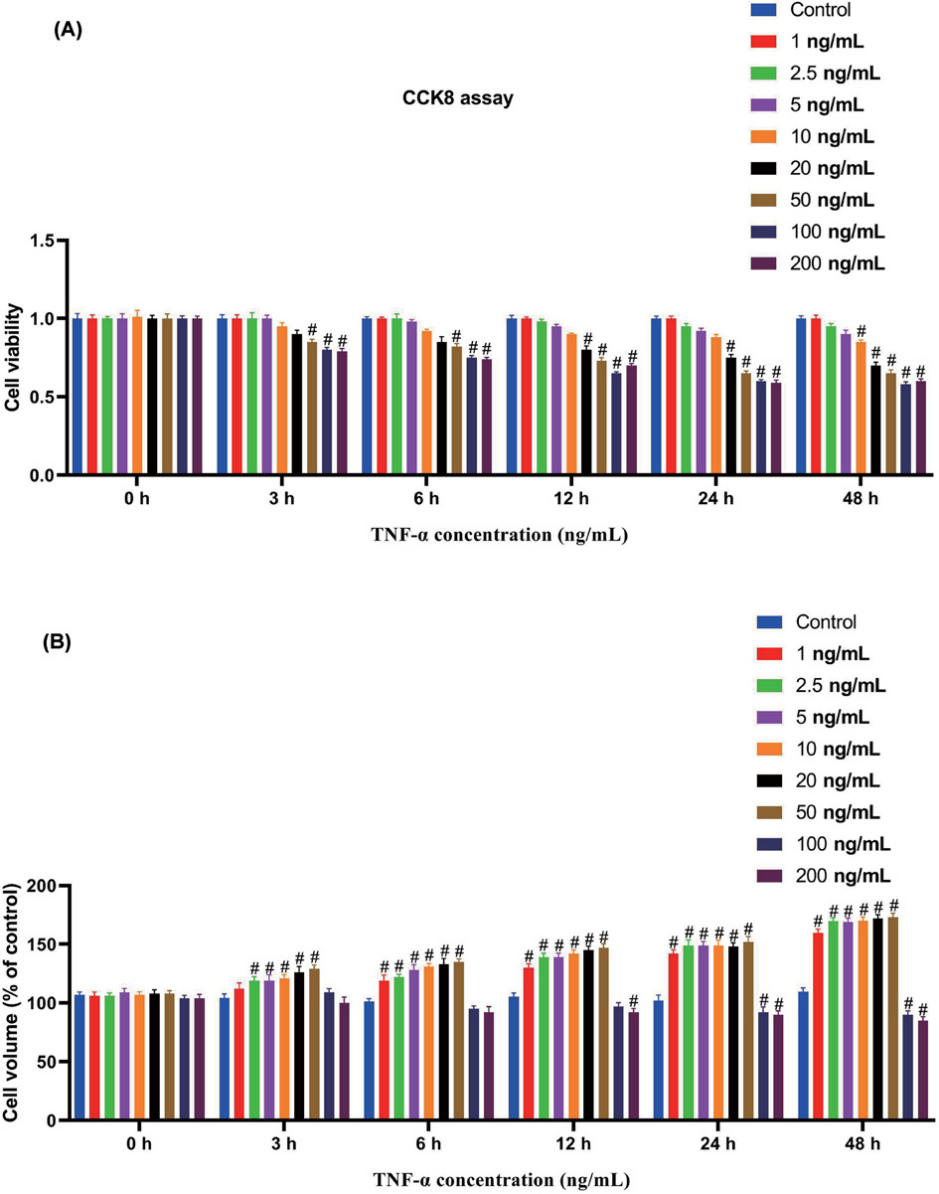
Detection of cell viability and cell volume of astrocytes treated with different concentrations of TNF-α (**A**) Astrocytes treated with TNF-α with 0, 1, 2.5, 5, 10, 20, 50, 100, 200 at 0, 3, 6, 12, 24, and 48 h, and cell viability assessed with CCK-8. (**B**) Cell volumes of astrocytes treated with TNF-α with 0, 1, 2.5, 5, 10, 20, 50, 100, and 200 ng/ml, at 0, 3, 6, 12, 24, and 48 h. *n*=3. ^#^, *P*<0.05, compared with the control group (0 ng/ml). One-way ANOVA was used for comparison between multiple samples.

To explore the effect of TNF-α at different concentrations on astrocytes, astrocytes mentioned in the present study were treated with TNF-α, respectively, using 0, 1, 2.5, 5, 10, 20, 50, 100, and 200 ng/ml for 0, 3, 6, 12, 24, and 48 h. The viability of astrocytes at 50, 100, and 200 ng/ml of TNF-α was significantly reduced at 3 h after treatment (*P*<0.05). At 12 h, the astrocyte viability in the 20 ng/ml group started to drop significantly (*P*<0.05). At 48 h, the astrocyte viability at the concentration over 10 ng/ml was markedly reduced (*P*<0.05) (Figure 1A). Meanwhile, we detected the changes in the cell volume of astrocytes at the same conditions and found that at 3 h, the cell volume in the 2.5, 5, 10, 20, and 50 ng/ml groups increased substantially (*P*<0.05). At 6 h, the cell volume of the 1 ng/ml group also increased significantly (*P*<0.05), and until 48 h, the astrocyte volume of the 1, 2.5, 5, 10, 20 and 50 ng/ml groups were significantly higher than that in the control group (0 ng/ml) (*P*<0.05). The cell volume of cells in the 100 and 200 ng/ml groups after 24 h of treatment was significantly lower than that of the control group (0 ng/ml) (*P*<0.05) (Figure 1B). These results indicate that TNF-α can cause edema in astrocytes and increase the cell volume, however, the TNF-α treatment at 100 and 200 ng/ml caused cell rupture due to excessive swelling. Moreover, TNF-α at above 10 ng/ml could suppress astrocyte viability.

### TNF-α can induce high expression of AQP4

**Figure 2:**
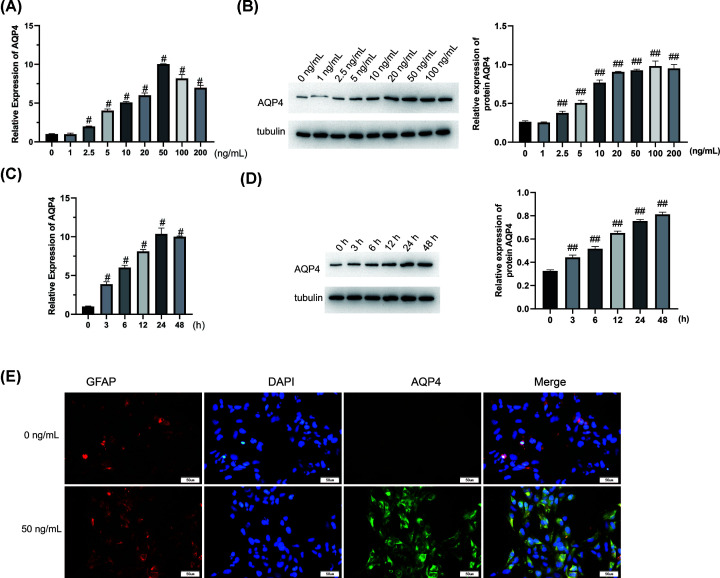
TNF-α can induce high expression of AQP4 (**A**) Astrocytes treated with TNF-α with 0, 1, 2.5, 5, 10, 20, 50, 100, and 200 ng/ml for 24 h, and qPCR was conducted to detect the expression of AQP4 mRNA. (**B**) Astrocytes treated with TNF-α with 0, 1, 2.5, 5, 10, 20, 50, 100, and 200 ng/ml for 24 h, and Western blotting was used to detect the expression of AQP4 protein. (**C**) After the cells were treated with 50 ng/ml TNF-α for 0, 3, 6, 12, 24, and 48 h, qPCR was used to detect the expression of AQP4 mRNA. (**D**) After the cells were treated with 50 ng/ml TNF-α for 0, 3, 6, 12, 24, and 48 h, the expression of AQP4 protein was detected by Western blot. (**E**) After the cells were treated with 50 ng/ml TNF-α for 24 h, cells were double-labeled with GFAP and AQP4 antibodies. Scale bar: 50 μm. *n*=3. ^#^, *P*<0.05; ^##^, *P*<0.01, compared with the control group (0 ng/ml). One-way ANOVA was used for comparison between multiple samples.

Next, to detect the influence of TNF-α on the expression of AQP4, we prepared astrocyte samples treated with TNF-α at 0, 1, 2.5, 5, 10, 20, 50, 100, and 200 ng/ml for 24 h, then extracted RNA and proteins to detect AQP4 expression. As shown in Figure 2A, the qPCR results showed that AQP4 mRNA expression was markedly increased following treatment with TNF-α at a concentration of 2.5 ng/ml or above (*P*<0.05). At the same time, AQP4 expression was the highest in the 50 ng/ml group. Western blot results were consistent with qPCR results (Figure 2B). We then proceeded to detect AQP4 expression in cells treated with 50 ng/ml TNF-α for 0, 3, 6, 12, 24, and 48 h. The expression level of AQP4 was the highest at 24 h treatment with 50 ng/ml TNF-α (Figure 2C,D). Furthermore, after the cells were treated with 50 ng/ml TNF-α for 24 h, they were double-labeled with antibodies against GFAP and AQP4. The astrocytes treated with 50 ng/ml TNF-α showed a significantly higher level of AQP4 protein than those in the control group (0 ng/ml), demonstrating that TNF-α-induced AQP4 are localized in astrocytes (Figure 2E).

### Regulatory effect of TNF-α on the NF-κB pathway

**Figure 3:**
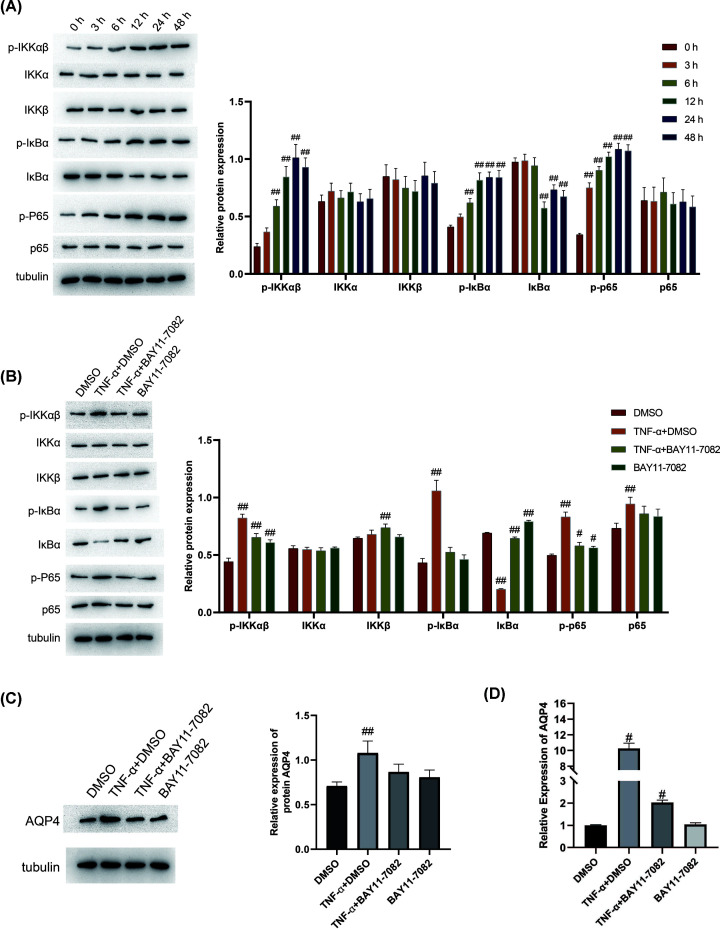
TNF-α activates the NF-κB pathway (**A**) A 50 ng/ml TNF-α was used to treat the cells for 0, 3, 6, 12, 24 and 48 h, Western blot was used to detect P-IKKα/β, IKKα, IKKβ, P-IκBα, IκBα, P-p65, and p65 expression. ^##^, *P*<0.01, compared with the control group (0 h). One-way ANOVA was used for comparison between multiple samples. (**B**) The NF-κB signaling pathway inhibitor BAY11-7082 or DMSO (negative control) was employed to pretreat primary astrocytes, then the cells were stimulated with TNF-α (50 ng/ml) for 24 h, and Western blot was subsequently used to detect P-IKKα/β, IKKα, IKKβ, P-IκBα, IκBα, P-p65, and p65 protein levels. ^#^, *P*<0.05; ^##^, *P*<0.01, compared with the control group (DMSO). One-way ANOVA was used for comparison between multiple samples. (**C**,**D**) NF-κB signaling pathway inhibitor BAY11-7082 or DMSO (negative control) was used to pretreat primary astrocytes, and then the cells were stimulated with TNF-α (50 ng/ml) for 24 h. Western blot and qPCR were used to detect AQP4 expression. *n*=3. ^#^, *P*<0.05; ^##^, *P*<0.01, compared with the control group (DMSO). One-way ANOVA was used for comparison between multiple samples.

To examine the effect of TNF-α on the NF-κB pathway, the cells were treated with 50 ng/ml TNF-α for 0, 3, 6, 12, 24, and 48 h, and the expressions of P-IKKα/β, IKKα, IKKβ, P-IκBα, IκBα, P-p65, and p65 were detected by Western blotting. The expressions of P-IKKα/β, P-IκBα and P-p65 increased significantly after TNF-α treatment, in addition, with the increase in the time of incubation, the content of these phosphorylated proteins also increased (Figure 3A).

To further verify the changes in the NF-κB signaling pathway, the primary astrocytes were treated with the NF-κB signaling pathway inhibitor BAY 11-7082 or DMSO (negative control), and TNF-α (50 ng/ml) to stimulate the cells for 24 h, and Western blotting was performed to detect the expression levels of the components of the NF-κB signaling pathway, P-IKKα/β, IKKα, IKKβ, P-IκBα, IκBα, P-p65, and p65. The expression levels of P-IKKα/β, P-IκBα and P-p65 did not change significantly in the cells treated with the inhibitor BAY 11-7082 prior to TNF-α treatment (Figure 3B). Additionally, after inhibition of the NF-κB pathway and stimulation of the cells with TNF-α (50 ng/ml), the expression of AQP4 was significantly lower than that in the TNF-α+DMSO group (Figure 3C,D). Based on these observations, it is clear that TNF-α exerts its influence on AQP4 expression in the astrocytes through the NF-κB pathway.

### TNF-α promotes transcriptional activation of AQP4

**Figure 4:**
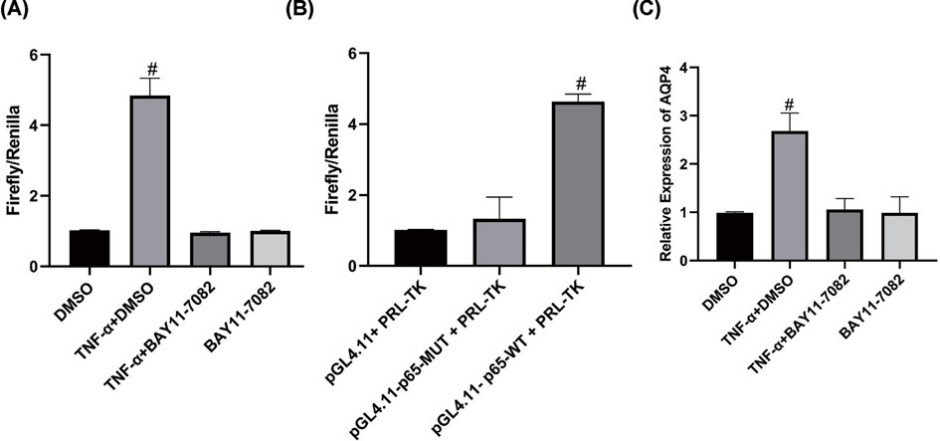
TNF-α promotes p65-mediated transcriptional activation of AQP4 (**A**) AQP4 promoter activity was determined by dual luciferase assay. ^#^, *P*<0.05, compared with DMSO group. One-way ANOVA was used for comparison between multiple samples. (**B**) AQP4 promoter with its p65-binding site mutated, the dual luciferase reporter system was used to detect the activity of the mutated AQP4 promoter. ^#^, *P*<0.05, compared with pGL4.11-p65-MUT+PRL-TK group. One-way ANOVA was used for comparison between multiple samples. (**C**) Following ChIP, qPCR was employed to detect the relative contents of AQP4 promoter DNA in the co-precipitation product. *n*=3. ^#^, *P*<0.05, compared with DMSO group. One-way ANOVA was used for comparison between multiple samples.

To elucidate the molecular regulation mechanism of TNF-α and NF-κB pathways on AQP4, we constructed a reporter gene plasmid in which firefly luciferase reporter gene linked to the promoter sequence of AQP4 gene. After the luciferase reporter gene plasmid was transfected into primary astrocytes, the cells were stimulated with TNF-α. Then the luciferase gene activity was measured for each treatment group. The results revealed that after the addition of TNF-α, the firefly/*Renilla* ratio increased (*P*<0.05), indicating that the AQP4 promoter activity increased, and transcription was enhanced. In cells to which the NF-κB signaling pathway inhibitor BAY 11-7082 was added, the ratio of firefly/*Renilla* did not differ from that of the DMSO group (Figure 4A).

After transfecting primary astrocytes with a luciferase reporter gene plasmid or a control luciferase reporter gene plasmid linked to the AQP4 gene promoter sequence but with mutations in the p65-binding site, the cells were stimulated with TNF-α. The results revealed that there was no significant difference between firefly/*Renilla* in the mutant group and the control group, while firefly/*Renilla* in the wildtype group increased significantly (*P*<0.05) (Figure 4B). Furthermore, the primary astrocytes were pretreated with the NF-κB signaling pathway inhibitor BAY 11-7082 or DMSO, and then stimulated with TNF-α. Cells from different treatment groups were collected and then ChIP assay was performed to detect the binding of p65 to AQP4 gene promoter in primary astrocytes. As illustrated in Figure 4C, the results revealed that p65 protein bound to AQP4 promoter, and the binding was better after TNF-α treatment, and BAY 11-7082 inhibited the binding that occurred in the presence of TNF-α.

### The effect of AQP4 and p65 on astrocytes

**Figure 5:**
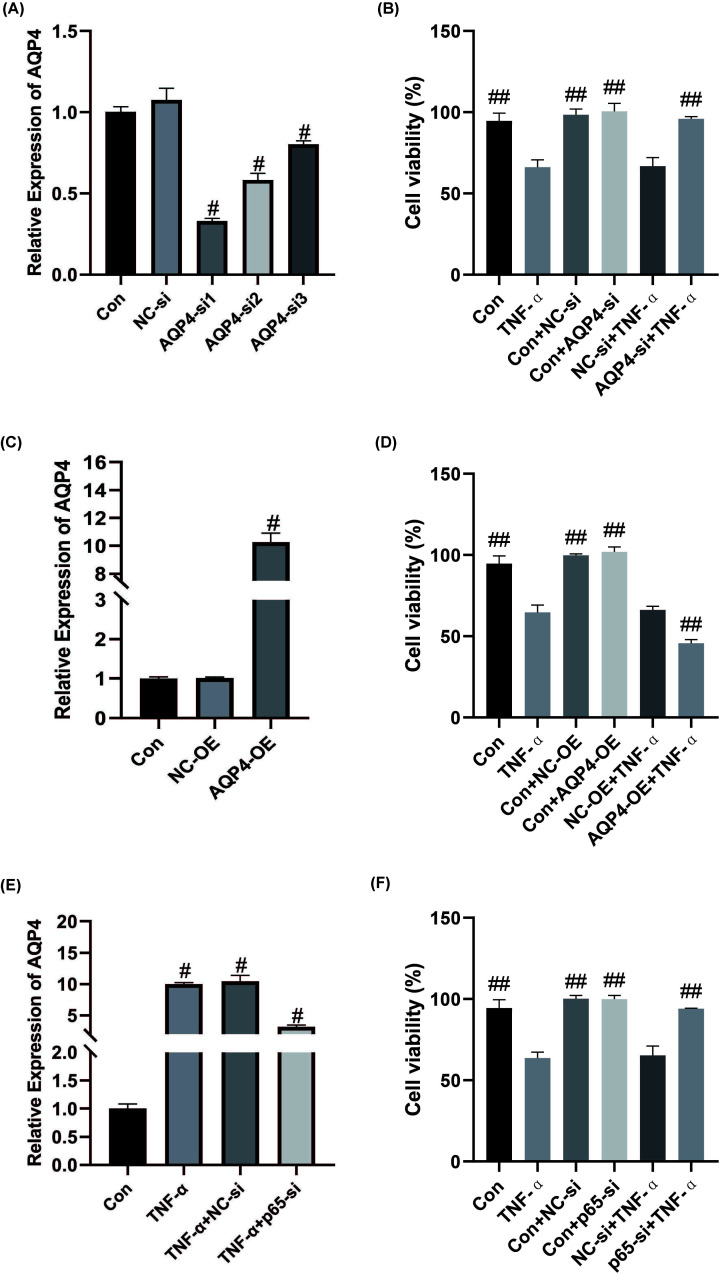
The effect of AQP4 and p65 on astrocytes (**A**) After interfering with AQP4 expression, qPCR was employed to detect the expression of AQP4. ^#^, *P*<0.05, compared with the NC-si group. (**B**) After interfering with the expression of AQP4, 50 ng/ml TNF-α was added for stimulation and CCK-8 was performed to detect cell viability. ^##^, *P*<0.01, compared with the TNF-α group. (**C**) After overexpression of AQP4, qPCR was performed to detect the expression of AQP4. ^#^, *P*<0.05, compared with the NC-OE group. (**D**) After overexpression of AQP4, 50 ng/ml TNF-α was added for stimulation and CCK-8 was conducted to detect cell viability. ^##^, *P*<0.01, compared with the TNF-α group. (**E**) After interfering p65 expression, qPCR was employed to detect the expression of p65. ^#^, *P*<0.05, compared with the control group. (**F**) After interfering p65, 50 ng/ml TNF-α was added for stimulation and CCK-8 was performed to detect cell viability. *n*=3. ^##^, *P*<0.01, compared with the TNF-α group.

Primary astrocytes were transfected with lentivirus vector for interference or overexpression of AQP4, and 50 ng/ml TNF-α was added to stimulate the transfected astrocytes. CCK-8 assay was performed to assess cell viability and the results indicated that after adding TNF-α, the cell viability of the interference AQP4 group was significantly higher than that of the TNF-α only group (*P*<0.05) (Figure 5A,B), while the cell viability of the AQP4 overexpression group was significantly lower than that of the TNF-α only group (Figure 5C,D). Moreover, the primary astrocytes were transfected with p65-interfered lentivirus or control lentivirus, and the astrocytes after transfection were stimulated with TNF-α. The results revealed that after interference with p65, the expression level of AQP4 mRNA was significantly reduced, and the astrocyte viability was significantly higher than that in the TNF-α group only (*P*<0.05) (Figure 5E,F). These results further demonstrate that TNF-α regulates the expression of AQP4 through the NF-κB pathway by p65 binding to AQP4 promoter and affects the viability of astrocytes.

## Discussion

The present study attempted to determine the effects of TNF-α on astrocyte viability and edema, and to reveal the regulatory mechanism of the NF-κB signaling pathway on AQP4 protein expression. Firstly, astrocytes were treated with TNF-α respectively using 0, 1, 2.5, 5, 10, 20, 50, 100, and 200 ng/ml for 0, 3, 6, 12, 24, and 48 h, and the results indicated that 50 ng/ml TNF-α treatment had the most obvious effect. Secondly, the present study demonstrated that TNF-α can induce the expression of AQP4, and increase the expression of P-IKKα/β, P-IκBα and P-p65, suggesting an activation of the NF-κB signaling pathway by TNF-α. Finally, by adding the NF-κB signaling pathway inhibitor BAY 11-7082 and performing interference experiments on AQP4 and p65, the regulatory mechanism of TNF-α on AQP4 was further confirmed, upon activation of the NF-κB signaling pathway by TNF-α, p65 binds to the promoter region of AQP4 gene and activates its transcription.

The main pathophysiological changes in cerebral hemorrhage are the formation of hematoma and secondary brain injury. Cerebral hemorrhage causes secondary brain injury through acute inflammation, the release of ROS, the destruction of the blood–brain barrier, and brain edema. Cerebral edema is an important factor affecting the prognosis of patients. AQP4 is a protein located on the cell membrane of astrocytes. It plays a vital role in controlling the water content in the cell. It is mainly distributed in astrocytes, ependyma, supraoptic nucleus, and paraoptic nucleus of hypothalamus. Many studies have shown that AQP4 is involved in the pathological process of cerebral edema [21,22]. In the cerebral edema caused by water intoxication and stroke, the brain water content of AQP4 gene knockout rats was significantly decreased, and the prognosis was better, indicating that down-regulation of AQP4 expression can be used as a way of treating cerebral edema [23,24]. In the present study, we found that after adding TNF-α, AQP4 expression increased significantly, while astrocyte viability decreased and cell volume increased, all of which are consistent with previous studies. In addition, in astrocytes cultured *in vitro*, after interfering with the expression of AQP4, it was found that the cell viability was significantly restored.

A variety of inflammatory factors, such as TNF-α and IL-1β, are expressed around the hemorrhage area after cerebral hemorrhage. TNF-α is recognized as a triggering factor in the immune response of cerebral edema secondary to cerebral hemorrhage. TNF-α can be first expressed on astrocytes, and then massively released, TNF-α can then directly or indirectly act on neurons or glial cells, induce the expression of IL-6, IL-1, IL-8 and other cytokines [25,26]. The study found that in an *in vitro* culture of astrocytes with TNF-α and IL-1β, compared with the control group, the AQP4 level of the TNF-α and I-1β intervention group was significantly increased. This result is consistent with the results from our study since we found that treatment with TNF-α at 2.5 ng/ml or above could result in a considerable increase in the expression of AQP4 mRNA, with the maximum expression in the 50 ng/ml group.

Previous studies have shown that NF-κB is an important factor for secondary damage after intracerebral hemorrhage. It can stimulate the expression of cytokines such as TNF-α and IL-1β. At the same time, these cytokines activate NF-κB again. Only activated NF-κB can be transported to the nucleus, causing a sustained or amplified inflammatory response. Therefore, the occurrence and development of cerebral hemorrhage is closely related to NF-κB availability. The use of TNF-α inhibitors can effectively control the activation of NF-κB, and can be a new method for treating cerebral hemorrhage [8,9,27,28]. In this study, adding 50 ng/ml TNF-α to treat cells for 0, 3, 6, 12, 24, and 48 h, led to a significant increase in the expression of IKKα/β, P-IκBα and P-p65, demonstrating that TNF-α can activate the NF-κB pathway. With the increase in the time of incubation, the levels of phosphorylated proteins also increased. Activation of NF-кB has been strongly associated with the stimulation of numerous ion channels/exchangers under diverse situations and with post-traumatic astrocyte swelling/brain edema regulation. A comparable study revealed a considerable increase in NKCC activity following trauma to cultured astrocytes, which was reduced by the NF-кB inhibitor BAY-11 7082, implying that NF-кB-mediated cell swelling following trauma is partially a result of increased NKCC activity [29]. Similarly, in our study, the NF-κB signaling pathway inhibitor BAY 11-7082 was used to pretreat primary astrocytes and then they were stimulated with TNF-α. The results revealed that the expressions of P-IKKα/β, P-IκBα and P-p65 did not change significantly after adding the inhibitor BAY 11-7082 to the cells and followed by TNF-α treatment. In addition, after the NF-κB pathway was inhibited, the cells were stimulated with TNF-α (50 ng/ml), and the expression of AQP4 was significantly lower than that of the cells treated with TNF-α alone. The present study established that TNF-α exerts its regulatory effect on AQP4 expression thereby affecting astrocytes via the NF-κB pathway.

## Conclusion

The results of the present study indicate that TNF-α activates the NF-κB pathway, promotes the binding of p65 to the AQP4 promoter region to enhance its transcription, ultimately reducing astrocyte viability thereby causing cell edema.

## Supplementary Material

Supplementary Figure S1

## Data Availability

All data are available upon request.

## Abbreviations

AQP4: aquaporin 4
BSA: bovine serum albumin
CCK-8: cell counting kit-8
ChIP: chromatin immunoprecipitation
DMSO: dimethylsulfoxide
GFAP: glial fibrillary acidic protein
IκB: inhibitor of κB
NF-κB: nuclear factor κ-light-chain-enhancer of activated B cells
PBS: phosphate-buffered saline
ROS: reactive oxygen species
SDS: sodium dodecyl sulfate
TNF-α: tumor necrosis factor-α
