# Supplementary figures and images for: TNF-α induces AQP4 overexpression in astrocytes through the NF-κB pathway causing cellular edema and apoptosis
Source: Biosci Rep. 2022 Mar 17;42(3):BSR20212224. doi: 10.1042/BSR20212224 (PMC8935387; doi:10.1042/BSR20212224)

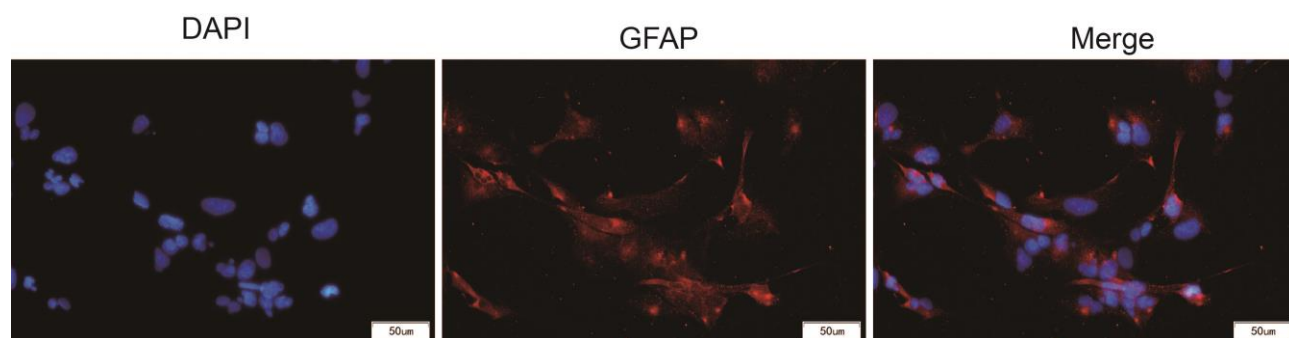

**Figure S1.** Cultured cells were stained with GFAP. Scale bar: 50  $\mu\text{m}$ .

Supplement: Supplementary Figure S1 [file BSR-2021-2224_supp.pdf]
